# Multi-modal imaging for the detection of early keratoconus: a narrative review

**DOI:** 10.1186/s40662-024-00386-1

**Published:** 2024-05-11

**Authors:** Muawyah Al Bdour, Hashem M. Sabbagh, Hisham M. Jammal

**Affiliations:** 1 Department of Ophthalmology, School of Medicine, The University of Jordan, Amman, Jordan; 2 The National Center for Diabetes Endocrinology and Genetics (NCDEG), Amman, Jordan; 3 Department of Ophthalmology, Faculty of Medicine, Jordan University of Science and Technology, PO Box 3030, Irbid, 22110 Jordan

**Keywords:** Keratoconus, Early, Suspect, Forme fruste, Subclinical, Multimodal, Imaging

## Abstract

Keratoconus is a common progressive corneal disorder that can be associated with significant ocular morbidity. Various corneal imaging techniques have been used for the diagnosis of established cases. However, in the early stages of the disease, which include subclinical keratoconus and forme fruste keratoconus, detection of such cases can be challenging. The importance of detecting such cases is very important because early intervention can halt disease progression, improve visual outcomes and prevent postrefractive surgery ectasia associated with performing corneal refractive procedures in such patients. This narrative review aimed to examine several established and evolving imaging techniques for the detection of early cases of keratoconus. The utilization of combinations of these techniques may further increase their diagnostic ability.

## Background

Keratoconus (KC) is a pathological condition characterized by the progressive thinning and steepening of the central or paracentral cornea, resulting in irregular astigmatism and visual impairment [1]. Both the prevalence of KC (ranging from 0.2 to 4790 cases per 100,000) and the incidence of this condition (at 1.5 to 25 cases per 100,000 per year) are variable throughout the world, with the Middle East having the highest occurrence [2, 3]. It is often diagnosed in people between the ages of 20 and 30 years and stabilizes by the fourth decade of life, with no significant sex variations in the frequency of the disease [4].

Histopathologically, this condition is characterized by epithelial thinning, basal cell degeneration, iron deposits, and cracks in the anterior limiting lamina. The stroma undergoes various changes, including central or paracentral stromal thinning, ectasia, lamellar reduction, and a changed collagen appearance [2]. Genetic factors (VSX1 and SOD1) and biomechanical weakening (proteoglycan degradation, collagen changes) are also involved [2]. Risk factors include eye rubbing, allergies, a family history of KC, parental consanguinity, Down syndrome, and connective tissue disorders [2, 3]. Most cases of KC are bilateral although asymmetry is sometimes observed. It is worth noting that there can be a significant delay of several years between the initial diagnosis of KC in one eye and the manifestation of the condition in the other [5].

A range of terminologies have been employed to delineate the phases of the ailment [6–8]. Clinical KC is distinguished by the presence of slit-lamp observations indicative of KC and positive results obtained from topography assessments. The term subclinical keratoconus (SCKC) denotes an eye that exhibits early topographic or tomographic indications of KC, and no clinical signs on slit lamp or retinoscopy examinations were observed, along with the presence of KC in the other eye, whereas KC suspect is defined as eyes with completely normal clinical examination and subtle topographic changes that are not completely compatible with definite KC pattern [7]. Forme fruste keratoconus (FFKC) is used to describe an ocular condition characterized by an apparently normal corneal topography and slit-lamp examination, while the other eye has KC. According to Klyce, the term KC suspect is reserved for corneas with subtle signs of KC (such as a localized area of abnormal steepening which is often inferior, or an asymmetrical, truncated or skewed-axis bowtie) but without evidence of clinical KC in either eye [9].

Early signs of KC include displacement of the cornea’s thinnest region from its central position [10], elevation of the posterior cornea [11], changes in the distribution of corneal epithelial cells [12], and changes in corneal aberrations, mainly vertical and total coma [13]. Various instruments and techniques have been utilized to identify these initial signs; nevertheless, each method has distinct strengths and weaknesses. The optimal use of these modalities is still subject to ongoing consensus formation.

The timely identification of KC during its initial phases is of utmost importance, as delayed detection can diminish the efficacy of some therapeutic approaches, including spectacles and contact lenses. In addition, it is advisable to administer therapies such as corneal cross-linking at the earliest opportunity to mitigate the risk of additional corneal deformity and reduce the need for corneal transplantation [14]. In addition, the assessment of KC is crucial in evaluating individuals who are being considered for refractive surgery, as there is a potential risk of iatrogenic corneal ectasia [15]. This review aimed to comprehensively examine the currently utilized imaging techniques for the early detection of KC.

## Main text

### Methodology

A PubMed database search was performed. The search terms used included: “early keratoconus”, “subclinical keratoconus”, “forme fruste keratoconus”, “keratoconus suspect”, “topography”, “tomography”, “wavefront”, “biomechanics”, “in vivo confocal microscopy”, and “optical coherence elastography”. The search scope was restricted to scholarly publications published from 2015 to November 2023.

We used the following MeSH terms (keywords) in the title of the publications in the online search:

1. ((‘‘early keratoconus” OR “subclinical keratoconus” OR “forme fruste keratoconus” OR ‘’keratoconus suspect”));
2. AND ((“topography” OR “tomography” OR “wavefront” OR “biomechanics” OR “confocal microscopy” OR “optical coherence elastography”));
3. AND ((analys*) OR (assess*) OR (detect*) OR (diagnos*) OR (discover*) OR (examin*) OR (identif*) OR (interpret*) OR (investigat*) OR (screen*)).

### Curvature-based corneal topography

Curvature-based corneal topography systems that rely on reflection are the most established and longstanding corneal imaging technologies currently available [16]. Contemporary corneal topographers or keratoscopes that rely on Placido-disc use images of concentric black and white rings reflected from the patient’s tear film. Computer algorithms then process these images to generate curvature maps of the anterior cornea, thereby quantifying relevant information.

Placido-disc-based corneal topography enables the generation of several indices that quantify surface abnormalities in the cornea. Specific indicators have been identified as having a high level of sensitivity in the diagnostic assessment of keratoconus [2]. The Rabinowitz-McDonnell test, which has been adjusted to have a keratometry value greater than 47.2 D and/or an inferior-superior (I-S) value above 1.4 D, has been documented as a method for detecting KC, with a demonstrated sensitivity of 96% [17]. The keratoconus prediction index (KPI) is a sensitive marker for detecting KC. It is generated by utilizing eight distinct topographic indices. A KPI greater than 0.23 has been found to have a sensitivity of 89% in identifying clinical cases of KC [18].

None of the above indices were explicitly established to identify individuals at risk for developing KC [18]. The utility of the keratoconus percentage index (KISA%), which is determined through the assessment of keratometry values, I-S values, relative skewing of the steepest radial axes (SRAX), and keratometric astigmatism (AST), has been demonstrated in identifying individuals who may have KC [18, 19]. A KISA% value above 100% indicates the presence of KC, while a KISA% value ranging from 60% to 100% suggests a suspicion of KC. Conversely, a KISA% value less than 60% is considered within the normal range. The keratoconus severity index (KSI), devised by Smolek and Klyce, employs ten distinct topographic indicators. The ability to predict individuals with suspected KC has been demonstrated, provided that the computed value is 15%–30% [19, 20].

The use of Placido-disc-based corneal topography comes with limitations, however. The current method exhibits limited coverage of the corneal surface [21], leading to the exclusion of vital data from the paracentral and peripheral corneal areas. Furthermore, this technique cannot offer insight into the posterior corneal surface, which has been identified as a site at which early changes occur during the progression of this illness [22]. Consequently, its effectiveness in detecting individuals with SCKC has been limited. The curvature-based corneal topography method assumes that the eye conforms to Gullstrand’s reduced eye model. However, this assumption might lead to erroneous identification of KC, as it may exhibit an asymmetric bowtie pattern in situations of normal eyes with an angle kappa above five degrees [22]. Moreover, certain corneal patterns caused by corneal scarring, dry eye, or the use of hard contact lenses may yield false positive results on videokeratoscopy like those seen in early KC. Similarly, a negative videokeratography may not show findings readily detectable by elevation-based Scheimpflug devices in early forms of KC [23].

### Elevation-based corneal tomography

Elevation-based corneal tomography devices are indispensable tools in the field of ophthalmology because they employ advanced techniques such as slit-scanning and Scheimpflug imaging. In slit-scanning, as demonstrated by the Orbscan, 40 light-slit projections are evenly distributed between the left and right scans and are aligned with the instrument axis, enabling a comprehensive evaluation of the cornea’s front and posterior surfaces. The SCORE software in the Orbscan machine utilizes the most discerning six parameters to calculate a KC risk score, where a score of zero is the cut-off point between a normal cornea (negative score) and a KC suspect (positive score). These parameters include pachymetry of the thinnest point, maximum posterior elevation in the central 3 mm, irregularity in the central 3 mm (diopters), vertical decentration of the thinnest point (mm), difference between mean central pachymetry and pachymetry of the thinnest point, and the I-S value (diopters) which is the difference between mean keratometric values of five points on the superior (S) and inferior (I) areas [24].

On the other hand, Scheimpflug imaging, utilized in devices such as the Pentacam, involves rotating the camera along the optic axis to capture light slits and reconstruct detailed corneal surfaces. Cutting-edge combination systems, including Orbscan II, Sirius, and Galilei, integrate slit-scanning or Scheimpflug technology with Placido reflection.

There are various advantages associated with elevation-based corneal topography compared to curvature-based systems. First and foremost, these systems employ measurement capabilities that operate at fast speeds, leading to enhanced levels of precision and repeatability when compared to reflection-based alternatives; hence, eye movements do not impact them which produces images of superior quality [22]. Furthermore, elevation-based tomography covers a considerably larger surface area of the cornea than does curvature-based corneal topography [21]. The increased measurement area provided by this expansion allows for improved detection of changes in the outside regions of the cornea, which are notably affected during the advanced phases of KC and pellucid marginal degeneration. Furthermore, it should be noted that elevation-based tomography does not rely on the premise that the eye is Gullstrand’s reduced eye, which is needed for videokeratography and other Placido-based topography methods, as previously described.

Another advantage of utilizing elevation-based topography is its ability to assess the posterior surface of the cornea. This characteristic is of particular significance due to the consistent findings of recent investigations, which have revealed a notable distinction in the posterior corneal surface between eyes in the initial phases of KC and those of healthy individuals. Even in SCKC patients, there is a significant disparity in posterior elevation compared to that in eyes that are considered normal [11, 25].

The Pentacam, Sirius, and Galilei topographers use various indicators to evaluate corneal shape and thickness comprehensively. The Belin-Ambrosio enhanced ectasia display (BAD) in the Pentacam, alongside BAD II and III, incorporates multivariate indices combining anterior and posterior elevation data with pachymetric data for thorough evaluation. Sirius employs measures such as the anterior (KVf) and posterior (KVb) keratoconus vertex, root mean square (RMS) and symmetry index of curvature, while Galilei introduces indices such as the asphericity asymmetry index (AAI), center/surround index (CSI), and differential sector index (DSI).

Shetty et al. compared several indices derived from these devices and reported the sensitivity and specificity of these devices for detecting SCKC [26]. The index of surface variance (ISV) and index of height asymmetry (IHA) in Pentacam, the CSI in Galilei, and the symmetry index front (SIf) in Sirius had high sensitivities for distinguishing SCKC from controls, whereas the IHA and curvature radius in Pentacam, the opposite sector index (OSI) in Galilei and the symmetry index back (SIb) in Sirius were the most specific indices for diagnosing subclinical cases.

In discriminating SKCN from normal eyes, Heidari et al. reported that Sirius SIb [sensitivity 86.2%, specificity 84.9%, area under the curve (AUC) 0.908], and Pentacam I-S value (sensitivity 80%, specificity 79.2%, AUC 0.862) followed by Pentacam random forest index (PRFI, sensitivity 71.1%, specificity 87.9%, AUC 0.847), and Corvis tomographic/biomechanical index (TBI, sensitivity 70.8%, specificity 83.0%, AUC 0.828) had the highest diagnostic ability [27]. Another report by Salman et al. also identified SIb as the best diagnostic parameter for detecting suspect KC with AUC of 0.86 [23].

Moreover, Koc et al. [28] used the Pentacam to find a statistically significant increase in corneal densitometry across many zones in eyes with SCKC compared to control eyes. The anterior layer in the 0 to 2 mm area had the best ability to distinguish SCKC from normal eyes. Their results suggest that heightened densitometry in the central zone may be a beneficial marker for identifying subclinical cases.

Golan et al. used the Galilei analyzer to examine and compare posterior corneal features between normal corneas and SCKC [11]. The results indicated that the maximum posterior elevation over the reference shape of the best-fit toric and aspheric surfaces exhibited the greatest discriminatory capacity. Elkitkat et al. also examined how well different Pentacam HR posterior elevation indices could detect early KC and how they are related to other factors [29]. The study examined posterior elevation from the best-fit sphere (BFS), the best-fit toric ellipsoid (BFTE), the exclusion map of Belin-Ambrosio’s display, and the difference map of BAD. The results showed that posterior elevation indices are sensitive early KC detectors, with posterior elevation from BFTE being the most sensitive.

Gharieb et al. used Sirius topography to find the best indices and cut-off values for revealing differences between thin normal corneas, FFKC, and early KC [30]. The assessment included the evaluation of keratometry indices, pachymetry indices, corneal aberrations, and elevation indices. The apex front curvature had the best discriminatory ability for early KC, whereas both the apex curvature and the coordinates characterized patients with FFKC. The KC summary indices exhibited high statistical significance in effectively differentiating between the three groups. The aberration characteristics of vertical coma and vertical trefoil were the most prominent in the study. Utilizing metrics such as the thinnest point elevation, RMS, and RMS/area proved to help distinguish between early KC and FFKC cases from thin and normal corneas.

Regarding the repeatability and agreement between different topography imaging devices, several studies assessed this issue and found high levels of repeatability and wide limits of agreement [31–33]. The difference in measurements between different devices were statistically significant, and therefore should not be used interchangeably. Additionally, the repeatability may be affected according to the status of the measured cornea (i.e., healthy corneas, KC severity, thin corneas) [34].

### Anterior segment optical coherence tomography

The advent of anterior segment optical coherence tomography (AS-OCT) in the early 2000s revolutionized the in vivo evaluation of corneal microstructure. Utilizing optical light scattering, AS-OCT generates high-resolution, cross-sectional images of the cornea, employing either time-domain optical coherence tomography (TD-OCT) or Fourier-domain optical coherence tomography (FD-OCT). While TD-OCT, exemplified by the Visante system, relies on a moving reference mirror, FD-OCT, exemplified by the RTVue, utilizes a fixed mirror and achieves significantly higher scanning rates, reaching 26,000 A-scans/second. Despite Visante’s slower speed, its longer wavelength (1310 nm) allows superior penetration of the sclera and iris. The RTVue, with a depth resolution of 5 μm, surpasses the Visante in speed and resolution, facilitating precise corneal assessments and direct evaluation of both anterior and posterior corneal power. As technology evolves, the cost-effectiveness of swept-source optical coherence tomography (SS-OCT), a type of FD-OCT, is expected to improve, marking continual progress in ophthalmic imaging capabilities.

Optical coherence tomography (OCT) has demonstrated efficacy in diagnosing KC [35, 36]. This diagnostic method relies on four parameters obtained from the pachymetry map’s core 5-mm-diameter region. The parameters included the minimum thickness of the cornea (Min), the difference between the minimum and maximum corneal thickness (Min-Max), the average variation in corneal thickness between the superonasal and inferotemporal (SN-IT) regions across rings of two to five diameters, and the pattern standard deviation (Std Dev) of the epithelial thickness [19].

Itoi et al. reported that anterior/posterior corneal surface areas (As/Ps) ratio measurements acquired from AS-OCT exhibited a high level of sensitivity and specificity when identifying FFKC (0.92 and 0.96 respectively, cut-off of 0.99), which was comparable to the BAD total deviation value (BAD-D) obtained by rotating Scheimpflug-based corneal tomography (1.00 and 0.90 respectively, cut-off of 1.33) [37]. Scuderi et al. also evaluated the ability of pachymetric indices obtained from spectral-domain optical coherence tomography (SD-OCT) to detect early KC [38]. C1–C2 had the highest sensitivity in detecting early cases of KC; C1 is the average corneal thickness at the points placed within a 1 mm diameter circle around the point with the lowest thickness, and C2 is the average corneal thickness at the points located diametrically opposite to the first one.

Furthermore, AS-OCT demonstrates superior accuracy compared to elevation-based imaging methods when scarring or corneal haze is present [39]. The instrument offers precise pachymetry mapping, while Orbscan II underestimates corneal thickness when central corneal scarring is present.

However, the main benefit of AS-OCT compared to elevation-based corneal examination in detecting early stage KC is its ability to visualize the epithelial layer comprehensively [40]. One proposed pathogenic change is a reduction in the density of the corneal basal epithelium. The degradation of basal epithelial cells causes destabilization of Bowman’s layer, leading to the eventual depletion of collagen fibrils in the anterior stroma [41, 42].

Ostadian et al. reported that in SCKC, there were notable differences in epithelial thickness, specifically, thickening in the inferior and temporal regions, compared to the overall corneal thickness. Additionally, patients with early KC exhibited a statistically significant decrease in the area characterized by minimal epithelial thickness [43]. Furthermore, SCKC has a unique epithelial donut pattern. As the progression of KC occurs, the epithelium gradually diminishes its capacity to conceal the steepness of the stromal layer, resulting in the emergence of irregular patterns characterized by numerous thin and thick regions [12, 44].

Li et al. employed the high axial resolution of the RTVue device, along with its noncontact epithelial thickness mapping capacity, to attain high diagnostic accuracy in detecting KC [45]. SCKC eyes exhibited distinctive patterns including superonasal epithelial thickening and inferotemporal epithelial thinning. In contrast, on the epithelial thickness map, the thickness of the normal eyes was most significantly greater at the center than at the other regions and gradually decreased toward the superior region.

The utilization of ultrahigh-resolution optical coherence tomography (UHR-OCT) enables the observation and quantification of the corneal epithelium and Bowman’s layer with high precision [46]. Xu et al. tested the power of vertical thickness profiles of the corneal epithelium and Bowman’s layer obtained by UHR-OCT to diagnose SCKC. The epithelial maximal ectasia index exhibited the most effective ability to distinguish SCKC from normal corneas.

Temstet et al. assessed how well OCT epithelial mapping improved the detection of FFKC [47]. Compared with normal corneas, FFKC corneas exhibit discernible attributes, such as a reduced epithelial thickness in the thinnest region of the cornea and an increased epithelial thickness in comparison to corneas affected by KC. Additionally, the epithelial thickness in the thinnest area of the cornea in patients with FFKC was found to be inferior, which was correlated with the region of minimal epithelial thickness and the highest posterior elevation. Their analysis showed good predictive accuracy of epithelial thickness in the thinnest corneal region, with a threshold of 52 μm for distinguishing between corneas with FFKC and those with normal corneas.

In their study, Yücekul et al. used Zeiss Cirrus 5000 HD SD-OCT, along with a recently constructed two-step decision tree based on previous research, to assess corneal and epithelial thickness maps in eyes categorized as normal, evident KC, or SCKC [48]. The two-step decision tree achieved a specificity of 100% and sensitivity of 100% in manifest KC, as well as a sensitivity of 90.4% in subclinical KC. The OCT pachymetric and epithelial map patterns showed a high level of agreement with the Belin-Ambrosio display of the Pentacam.

Salman et al. used high-definition SD-OCT to compare the diagnostic ability of corneal thickness and epithelial thickness maps in KC and suspect KC cases [49]. While all corneal and epithelial thickness variables assessed were successful in differentiating KC eyes from normal ones, none of these variables were able to differentiate suspect KC from normal eyes with high accuracy (AUC < 0.8 for all variables), with the highest diagnostic power for the minimum minus the maximum corneal epithelial thickness (Min-Max) in the paracentral 2–5 mm (AUC = 0.71; cut-off ≤ -9 μm) and the central corneal thickness (CCT) (AUC = 0.76; cut-off ≤ 533 μm).

The MS-39 is a corneal imaging device combining spectral-domain AS-OCT and Placido based topography. Separate corneal layers are detected with very high resolution and a wide field epithelial thickness map is encompassed, which is useful in the diagnosis of subclinical forms of KC in addition to topographic measurements of the anterior corneal surface. Similar to the anterior surface measurements obtained with Sirius, it produces corneal elevation data for the whole anterior segment [50].

Toprak et al. assessed the diagnostic values of corneal epithelial and stromal thickness distribution characteristics in FFKC and SCKC using the MS-39 and reported that FFKC cases had increased central epithelial/stromal ratio and asymmetric superior-nasal epithelial thinning compared to the control group. On the other hand, subclinical KC cases had higher epithelial/stromal ratios in the 5-mm temporal and superior zones compared to control cases [51].

Heideri et al. also evaluated the thicknesses of different corneal layers for identifying KC and SCKC using SD-OCT [52]. Total cornea and stroma in KC and SCKC, and epithelium in KC were significantly thinner compared to the control group. The highest AUC values were observed for CCT in KC (AUC 0.90) and SCKC (AUC 0.78). The diagnostic accuracy was significantly higher for stromal thickness in KC (AUC 0.87) and SCKC (AUC 0.75) than other individual corneal layers, indicating that central corneal stromal thinning was the most sensitive diagnostic index for early detection of SCKC.

The corneal epithelial resurfacing function is a process in which the corneal epithelium adapts to changes in the corneal surface to maintain a smooth and regular corneal surface by thickening over areas of flattening and thinning over areas of steepening and forms the basis for interpreting epithelial thickness maps. Conditions like chalazia, dryness, contact lens wear, and atypical keratoconic presentations may result in inaccurate interpretations of epithelial thickness maps since these conditions may cause central corneal flattening with corresponding epithelial thickening [53]. In the preoperative keratorefractive surgery assessment scenario, the ophthalmologist may incorrectly perceive that these patients are experiencing central focal thickening per the corneal resurfacing function, and therefore, reliance on epithelial thickness map alone may be insufficient for keratorefractive surgery screening in cases with early KC. In such cases, valuable tomographic parameters provided by other systems must be combined with epithelial thickness map for optimal patient management [54].

Polarization-sensitive optical coherence tomography (PS-OCT) is a new emerging modality, which besides providing anatomic data obtained by AS-OCT, evaluates corneal birefringence by measuring corneal phase retardation. The cornea is known to be optically birefringent due to the high organization of parallel collagen fibrils [55]. An advantage of using PS-OCT to measure corneal birefringence, is its high depth of resolution and high speed which reduces motion artifacts.

In KC, the arrangement of regular fibril lamellae is altered resulting in changes in corneal birefringence. In an earlier study, corneal phase retardation was reported to be sensitive to discriminate early KC [56]. Recent studies evaluated corneal phase retardation in healthy, thin, asymmetric KC, and clinical KC corneas, and showed higher phase retardation in clinical KC eyes [57, 58].

### Noncontact tonometry

The etiology and pathogenesis of KC are not yet fully understood, yet certain biochemical, cellular, and microstructural variations have been observed. Biochemical alterations include heightened enzymatic activity of proteolytic agents and a reduction in their inhibitory factors [59], as well as variations in the composition and arrangement of proteoglycans [60]. Corneal keratocytes demonstrate a gradual decrease, leading to disruption of the structural alignment of collagen fibers. The expected consequences of these alterations include the regulation and arrangement of structural elements in the cornea, which are likely to have a detrimental effect on structural integrity, resulting in atypical deformation of the cornea. Aberrations in biomechanical response between KC corneas and normal corneas have been observed in experimental investigations conducted on ex vivo samples [61, 62]. The occurrence of corneal geometric changes is regarded as a secondary indicator, whereby the initial modifications manifest in microstructures and biomechanical properties. Hence, it is imperative to understand the biomechanical properties of the cornea to detect early KC effectively.

The measurement of corneal biomechanics in vivo continues to present challenges as only two commercially available tools have been employed to aid in diagnosing KC. The ocular response analyzer (ORA), released in 2005, enables the assessment of the cornea’s biomechanical reaction in vivo [63]. It involves the application of an air pulse to induce a temporary indentation on the cornea, followed by the measurement of infrared reflectance. This process yields two discernible peaks: pressure 1 in the inner direction and pressure 2 in the outward direction. Corneal hysteresis (CH) is a parameter that quantifies the disparity between two pressures and serves as an indicator of corneal viscosity and the capacity to absorb energy [64]. The corneal resistance factor (CRF) is a measure that quantifies the general resistance of the cornea to deformation [63, 64]. In eyes with KC, there is a noticeable decrease in CH and the CRF, as indicated by lower values [65]. Additionally, both measurements are positively related to KC severity. This finding suggests that these conditions lead to a decrease in the ability of the cornea to dampen vibrations and overall resistance. Even so, depending exclusively on CH or the CRF may not consistently yield definitive differentiation between mild KC eyes and normal eyes [66].

The Corvis ST, released in 2010, is a noncontact device that offers insights into the corneal biomechanical response using dynamic Scheimpflug imaging analysis. The imaging system acquires approximately 140 cross-sectional images while inducing dynamic deformation by an air puff. These images are subsequently used to analyze ten deformation characteristics linked with the tissue’s mechanical stiffness [67]. The parameters of interest encompass temporal measurements, spatial dimensions, and corneal velocity observed during the initial applanation (AT1, AL1, and AV1), as well as the subsequent recovery leading to the second applanation (AT2, AL2, and AV2). Furthermore, other parameters are assessed, including the time needed to achieve the highest level of concavity, the distance between the peaks of the concave curve, the radius of the central concave curvature, and the amplitude of deformation (DA) at the point of maximum deformation [64]. In addition to the ten metrics provided by the device, the use of a high-speed Scheimpflug camera allows for precise observation of cross-sectional corneal deformation resulting from the application of air pressure.

The Corvis biomechanical index (CBI) and the combined parameter TBI have been developed as sophisticated parameters to address the constraints associated with the original corneal biomechanical measurements acquired by the Corvis ST dynamic Scheimpflug analyzer [68, 69]. The CBI is calculated using dynamic corneal deformation data and aims to improve the sensitivity of detecting initial manifestations of ectatic corneal disorders. This index offers a more reliable diagnostic tool for clinicians. However, integrating corneal tomography data from the Pentacam with biomechanical assessment data from the TBI yields a comprehensive index that enhances the precision of identifying ectatic corneal disease [68]. Both indices are significant breakthroughs in the area as they enhance the accuracy and efficiency of diagnostic procedures when studying corneal biomechanics and associated diseases.

Studying the ability of corneal biomechanical parameters to detect ectasia early, Sedaghat et al. reported that the characteristics of Corvis ST with the highest accuracy for discriminating normal corneas from SCKC corneas were the highest concavity radius (HCR), integrated radius (IR), deformation amplitude ratio (DAR), and TBI [70]. They also found that the CRF measured by ORA had a greater capacity for detection than did CH. Furthermore, Huo et al. reported notable differences in biomechanical metrics, such as A1-time and IR, between SCKC corneal biomechanics and those of FFKC corneal biomechanics [71].

Wallace et al. aimed to assess corneal biomechanical parameters (CBI), PRFI, and TBI in KC detection. The results indicated that the TBI and PRFI could be used to distinguish between normal corneas and those with asymmetrical corneal ectasia effectively [72]. However, their performance was not statistically superior to that of the CBI or BAD-D. The TBI outperformed the CBI and BAD-D in discriminating healthy corneas from fellow eyes without apparent ectasia, serving as a valuable indicator for identifying subclinical and clinically apparent KC. On the other hand, the Sirius SIb (AUC = 0.908) and Pentacam I-S difference value (AUC = 0.862) outperformed the PRFI (AUC = 0.847) and Corvis TBI (AUC = 0.820) in distinguishing SCKC from normal eyes [27]. 

Finally, Peyman et al. used Corvis ST parameters, including DA, TBI, CBI, Ambrósio relational thickness to the horizontal profile (ARTh), and stiffness parameter at the first applanation (SPA1), to distinguish between SCKC and normal eyes [73]. SCKC eyes displayed a statistically significant increase in DA ratio, TBI, and CBI compared to normal eyes. Conversely, the ARTh and SPA1 were significantly lower in SCKC eyes. The above parameters may be helpful in distinguishing normal eyes from eyes with SCKC.

### Brillouin light-scattering microscopy

Brillouin light-scattering microscopy, also known as Brillouin spectroscopy, was first introduced in the field of ophthalmology in 1980 [74]. It has since gained recognition as a noninvasive technique for assessing the mechanical properties of the cornea [75]. The utilization of the interaction between narrow-banded laser light and phonons in matter presents an opportunity to gain an extensive understanding of corneal biomechanics. Furthermore, they can improve the accuracy of corneal surgical procedures such as keratotomies and contribute to our understanding of the reliability of applanation tonometry [76]. Phonons, which are wavelets resulting from molecular vibrations, are found universally in tissues at room temperature. These wave packets propagate through matter at a velocity influenced by various parameters, including the elastic moduli. The relationship between the Brillouin frequency shift, which occurs during the interaction of laser light with phonons, and the velocity of these phonons is shown to be directly proportional. Consequently, the Brillouin frequency shift is also directly proportional to the square root of the elastic modulus. By measuring the Brillouin frequency shift in the cornea, one can gain noninvasive access to the bulk elastic modulus of the cornea [77].

Seiler et al. investigated Brillouin frequency shifts using Brillouin spectroscopy perpendicular to the corneal surface in both healthy individuals and those with KC [74]. The findings demonstrated a statistically significant association between age and the central Brillouin frequency shift, suggesting that the stiffness of normal corneas increases with age. Corneas affected by KC exhibit a notable decrease in the Brillouin frequency shift at the point of minimum thickness compared to unaffected corneas. The Brillouin frequency shift at the point of maximum posterior elevation correlates best with geometry-derived KC indices. Nevertheless, the Brillouin frequency shift examination revealed a diminished level of sensitivity and specificity compared to established techniques such as maximum keratometry (Kmax) and thinnest pachymetry when detecting KC.

In addition, Randleman et al. employed motion tracking (MT) Brillouin microscopy to analyze focal biomechanical alterations in patients with SCKC [78]. Scheimpflug tomography and a custom-built MT Brillouin microscopy imaging system were utilized to generate a range of metrics. The investigation computed the mean and minimum measurements of the MT Brillouin in the anterior plateau region (Plateau) and the anterior 150 μm (A150). The Scheimpflug metrics investigated in the study included the I-S value, Kmax, the thinnest corneal thickness (TCT), asymmetry indices, BAD-D, and the Ambrósio relational thickness maximum (ARTmax). Although there were no notable differences in age, sex, refraction, or visual acuity between the groups, the analysis of Scheimpflug metrics indicated significant disparities in the thinnest corneal thickness, I-S value, index of vertical asymmetry, and KC index. The MT Brillouin metrics, particularly the mean and minimum values in the anterior plateau region and anterior 150 μm, demonstrated evident disparities between the control and SCKC eyes, effectively distinguishing the two groups.

### Wavefront aberrations

Higher-order aberrations (HOAs) are measured using wavefront analysis, utilizing Zernike polynomials as a representation [22, 79]. HOAs play a crucial role in assessing the progression of KC and detecting first-order changes in the corneal surface, particularly in patients for whom SCKC is suspected. The initial investigations determined wavefront aberrations by analyzing surface height measurements obtained from videokeratography.

In their research, Schwiegerling and Greivenkamp showed that a composite index of two primary aberrations—defocus and astigmatism—can reliably indicate KC [79]. The accuracy of this index was comparable to that of traditional curvature characteristics, such as the I-S value, steepest radial axis, and surface asymmetry index. In a follow-up study, Gobbe and Guillon reported that eyes classified as KC suspects exhibit a notable elevation in vertical coma compared to normal eyes [80].

The Shack-Hartmann wavefront sensor has been widely adopted to evaluate wavefront properties in KC, SCKC, and FFKC patients. Vertical coma has been identified as the most significant HOA in eyes affected by KC [81]. Vertical coma was consistently identified as the anomaly that exhibited the most marked difference between patients with SCKC and normal eyes [82, 83]. However, vertical coma alone did not produce satisfactory sensitivity or specificity in distinguishing SCKC from normal eyes [84].

Heidari et al. compared the precision of the Pentacam, Sirius, and OPD-Scan III for distinguishing SCKC from normal corneas based on wavefront parameters [85]. The study identified key parameters – the front Baiocchi Calossi Versaci (BCV) index with Sirius, front vertical coma (Z3–Z1) with Pentacam, and corneal Z3–Z1 with OPD-Scan III – that showed significant value for distinguishing SCKC. Overall, the findings suggest that corneal wavefront indices calculated from these devices can be used to effectively distinguish between normal corneas and early KC, with the front BCV index calculated from Sirius showing the highest accuracy in diagnosing SCKC, followed by Z3–Z1 with the Pentacam and OPD-Scan III.

Moreover, a study by Naderan et al. revealed a significantly greater RMS of all ocular aberration measurements in KC and FFKC patients than in healthy individuals [13]. Corneal aberrations were notably greater in KC patients than in normal individuals, with only specific parameters showing significant differences between FFKC patients and normal individuals. Ocular vertical and total coma were the most productive at discriminating keratoconic eyes, while ocular total higher aberration and total coma were identified as key parameters for distinguishing FFKC eyes from normal eyes.

Castro-Luna and Pérez-Rueda aimed to describe the topographic, pachymetric, and aberrometric features of corneas in a normal subject and those in patients with KC and SCKC [86]. The objective of their study was to develop a diagnostic model for SCKC. The findings indicated notable variations in vertical asymmetry to 90° and CCT when comparing normal corneas to those in the early stages of KC. The diagnostic model, comprising minimal corneal thickness, anterior coma to 90°, and posterior coma to 90°, accurately identified SCKC in 98.18% of the patients, with a sensitivity of 97.59% and a specificity of 98.78%.

In their study, Salman et al. evaluated the use of anterior and posterior corneal aberrations in differentiating suspect KC from normal eyes, and found that only the anterior corneal HOAs, particularly coma (RMS coma 3, ± 1, AUC = 0.922; cut-off > 0.2) were of high value in detecting suspect KC [87].

### In vivo confocal microscopy (IVCM)

IVCM is a noninvasive imaging technique that quantitatively analyzes corneal cellular architecture in its natural state. Optical sectioning and confocal imaging are used to scan through the cornea, capturing cellular details such as the epithelium and stromal keratocytes. Despite the limited individual image size, automated methods create mosaic images for broader analysis. The laser scanning confocal microscope, the current commercial design, offers high magnification (800×), a lateral resolution of 1 μm, and an axial resolution of 4 μm.

Ozgurhan et al. used IVCM to study KC and reported decreased stromal keratocyte densities and larger stromal nerve diameters in affected individuals [88]. In addition, Pahuja et al. revealed significant differences in nerve density and length between affected and unaffected eyes [89].

Furthermore, Ghosh et al. investigated corneal cell morphology in KC patients and discovered qualitative variations in corneal cell morphology and statistically significant decreases in nerve fibers and keratocytes in affected eyes [90]. Finally, Flockerzi et al. reported a shorter corneal nerve fiber length and a distinct tortuous pattern in the subbasal nerve plexus from KC patients [91].

### Optical coherence elastography (OCE)

Within the domain of in vivo research, OCE integrates a loading mechanism for the application of stimulation forces alongside an OCT system to observe tissue displacements [92]. This synergistic approach facilitates the accurate assessment of biomechanical properties. The loading tactics utilized in OCE include a wide range of approaches, encompassing static and dynamic loading, active and passive loading, and contact and noncontact loading methodologies. Detection methods utilized in OCE include speckle tracking and phase-sensitive detection, each of which is employed in distinct phases of the elastography procedure. The three main approaches used in OCE for assessing corneal biomechanics in vivo include air-puff applanation, wave-based elasticity focusing on the shear modulus, and natural-frequency OCE, each of which presents challenges such as spatial resolution limitations, mitigation of eye movements, and careful consideration of influencing factors [92].

Lan et al. utilized a microliter air-pulse OCE to quantify the natural frequency of the human cornea in vivo [93]. Their study revealed oscillation magnitudes ranging from submicrometers to subnanometers and showed superior repeatability and reproducibility compared to alternative OCE techniques. Moreover, Crespo et al. employed audio-sound frequencies to induce and evaluate corneal resonant responses and subsequently determined Young’s moduli by applying an empirical equation [94].

Research by De Stefano et al. utilized the OCE to investigate the depth-specific biomechanical characteristics of healthy and keratoconic eyes [95]. This pioneering study marked the first application of OCE in individuals with KC, providing a practical biomarker for altered stromal stiffness gradients in keratoconic eyes. Employing a custom swept-source OCE device, the researchers induced corneal perturbation to examine displacements in the anterior and posterior stromal layers, revealing a significant reduction in anterior stromal stiffness in the KC. The introduction and validation of the biomechanical property ratio (Ka/Kp) proved efficient in distinguishing between normal and keratoconic eyes.

### Artificial intelligence (AI)

Although multimodal imaging of the cornea remains the mainstay method of diagnosing early KC, AI, with its complex algorithms and rapidly advancing improvements, has been recently investigated and utilized in enhancing the diagnostic accuracy of KC. Several authors reported on the use of AI in differentiating KC from normal eyes, SCKC from normal eyes, and classification of the severity of the disease [19, 96–98].

AI depends on the specific imaging device that it is applied to, the number and type (images vs. data) of parameters used alone or in combination, and whether single or multiple algorithms are used. Several algorithms are used in AI, with neural networks (NNs), naïve bayes (NB), and random forest (RF) being the most used. A recent systematic review and meta-analysis found that NNs followed by NB had the highest sensitivity and specificity in detecting clinical and SCKC [99]. The most commonly used imaging device was the Pentacam.

Output generated by AI models from one imaging device is not interchangeable with that from a different device due to different inputs. Additionally, selecting the appropriate inputs by the examiner for each AI model can improve the accuracy of the model.

In another systematic review, AI showed good accuracy, with a summary sensitivity of 90.0% and a summary specificity of 95.5% for detecting subclinical KC, which was lower than that for clinical KC (a summary sensitivity of 98.6% and a summary specificity of 98.3%). However, the review reported high risk of bias, unexplained heterogeneity of the results, and high applicability concerns in the reviewed studies [100].

### Genetic screening for keratoconus

Considering the role of genetic factors in keratoconus, several studies demonstrated various genetic abnormalities, involving VSX1, TGFBI, LOX, COL5A1, and SOD1 [101]. Moreover, asymptomatic relatives of KC patients showed findings consistent with FFKC on the BAD of Pentacam corneal tomography, along with co-segregated gene variants, implying that genetic testing may be used to identify family members with forme fruste disease [102].

Recently, genetic testing that assesses an individual’s risk of KC by testing for 75 genes associated with KC, has been developed by AvaGen™ (Avellino Lab USA Inc., Menlo Park, CA) [103]. This is a rapid test conducted using a buccal swab, and the results may be combined with other corneal imaging indices (cross-sectional and longitudinal), to improve the accuracy of AI to diagnose early KC cases and predict future disease progression, and thus help to identify eyes that may benefit from early intervention.

Table 1 summarizes the various indices and their anatomical locations in the discussed imaging modalities described in this review for the detection of established and early keratoconus.

**Table 1:** Modalities and indices used for diagnosing established and early keratoconus and the corresponding anatomical location

Imaging modality	Indices for KC diagnosis	Indices for early KC detection	Corneal anatomy
Reflection-based topography	K-value, I-S value, KPI, KISA%, KSI	-	Anterior
Elevation-based tomography	Anterior and posterior elevation of the corneal apex, keratometric values, pachymetric data based on comparing reconstruction of the anterior and posterior corneal surface to a best-fit sphere	ISV, IHA, BAD-D, PRFI, I-S value (Pentacam), CSI (Galilei), SIb, KVf (Sirius) Corneal densitometry (The anterior layer in the 0 to 2 mm area) PE from BFTE	Anterior/stromal/posterior
Anterior segment optical coherence tomography	Min, Min-Max, SN-IT, and Std Dev	Pachymetric indices C1–C2 Epithelial donut pattern Superonasal epithelial thickening and inferotemporal epithelial thinning The vertical maximum ectasia index of epithelium and horizontal maximum ectasia index of Bowman’s layer Epithelial thickness in the thinnest corneal zone Increased central epithelial/stromal (E/S) ratio CCT As/Ps Epithelial PSD	Anterior/epithelial/stromal/posterior
Noncontact tonometry	CH and CRF with ORA Radius value of central concave curvature at highest concavity, central corneal thickness, CBI, and TBI with the Corvis ST	TBI, CBI, HCR, IR, and DAR	Stromal
Brillouin light-scattering microscopy	Brillouin frequency shift	The mean and minimum values in the anterior plateau region and anterior 150 microns	Stromal
Wavefront aberrations	Vertical coma, total coma, total HOAs, front/back Zernike coefficient Z3–1	Vertical coma, BCVf, ocular total HOA, total coma Minimal corneal thickness, anterior coma to 90°, and posterior coma to 90°	-
In vivo confocal microscopy	Anterior and posterior stromal keratocyte density, epithelial cell shape, corneal nerve fiber length, subbasal nerve density, and stromal nerve diameter	-	Epithelial/stromal/endothelial
Optical coherence elastography	Ka/Kp	-	Stromal

*K-value* = keratometry value; *I-S value = *inferior-superior value; *KPI* = keratoconus prediction index; *KISA*% = keratoconus percentage index; *KSI* = keratoconus severity index; *ISV* = index of surface variance; *IHA* = index of height asymmetry; *BAD-D* = Belin-Ambrósio enhanced ectasia display deviation; *PRFI* = Pentacam random forest index; *CSI* = center/surround index; *SIb* = symmetry index back; *KVf* = keratoconus vertex front; *PE* = posterior elevation; *BFTE* = best-fit toric ellipsoid; *Min* = minimum corneal epithelial thickness; *Min-Max* = minimum minus maximum corneal epithelial thickness; *SN-IT* = superonasal-inferotemporal; *Std Dev* = standard deviation; *C1* = average corneal thickness at the points placed within a 1 mm diameter circle around the point with the lowest thickness, *C2* = the average corneal thickness at the points located diametrically opposite to the first one; *C1-C2* = the difference between C1 and C2; *CCT* = central corneal thickness; *As/Ps* = anterior/posterior corneal surface areas ratio; *PSD* = pattern standard deviation; *CH* = corneal hysteresis; *CRF* = corneal resistance factor; *ORA* = ocular response analyzer; *CBI* = Corvis biomechanical index; *TBI* = tomographic/biomechanical index; *HCR* = highest concavity radius; *IR* = integrated radius; *DAR* = deformation amplitude ratio; *HOAs* = higher-order aberrations; *BCVf* = Baiocchi Calossi Versaci front; *Ka/Kp* = slope of the force/displacement function for anterior (a) and posterior (p) corneal stromal regions

Table 2 summarizes the best parameters (highest AUROC) differentiating early keratoconus (FFKC, SCKC, KC suspect) from normal eyes, along with the AUROC, sensitivity, specificity, and cut-off values.

**Table 2:** The best parameters (highest AUROC) differentiating early keratoconus (FFKC, SCKC, KC suspect) from normal eyes, along with the AUROC, sensitivity, specificity, and cut-off values

Author	Year	Modality	Parameter with highest AUROC	AUROC (%)	Cut-off value	Sensitivity (%)	Specificity (%)
**FFKC vs. Normal**							
Temstet [47]	2015	AS-OCT (RTVue)	Epithelial thickness in the thinnest corneal zone	79.5	52	88.9	59.5
Naderan [13]	2018	Aberrometry (OPD-Scan II)	Ocular total HOA	90.8	0.306	91.7	95.3
Itoi [37]	2020	AS-OCT (CASIA)	As/Ps	98	0.99	92	96
Thulasides [10]	2020	Rotating Scheimpflug (Pentacam)	BAD-D	85.9	0.835	93.3	32.4
Elkitkat [29]	2021	Rotating Scheimpflug (Pentacam)	PE from BFTE	98.9	> 4	96	96.15
Toprak [51]	2021	AS-OCT (MS-39)	Epith/Stromal thickness ratio	NA	NA	75	94.3
Sedaghat [70]	2023	NCT/Scheimpflug (Corvis ST)	TBI	96.6	NA	NA	NA
Gharieb [30]	2024	Rotating Scheimpflug (Sirius)	KVf	83.1	NA	NA	NA
**SCKC** **vs.** **Normal**							
Li [45]	2016	AS-OCT (RTVue)	Epithelial PSD	98.5	> 0.041	96	100
Xu [46]	2016	UHR-OCT (Custom-built)	EEI-MAX and BEI-MAX	97	NA	91	93
Shetty [26]	2017	Rotating Scheimpflug (Pentacam)	BAD-D	88.7	> 1.6	83.8	86
Golan [11]	2018	Rotating Scheimpflug (Galilei)	MPE at BFTA	87.7	11.5	79.5	84.8
Koc [28]	2018	Rotating Scheimpflug (Pentacam)	Corneal densitometry (anterior layer, 0–2 zone)	88.3	19.7	75	90
Castro-Luna [86]	2020	Rotating Scheimpflug (Pentacam)	MCT, anterior & posterior coma to 90°	92	NA	75	96.34
Heidari [85]	2020	Rotating Scheimpflug (Sirius)	BCVf	87.7	NA	87.7	83
Heidari [27]	2021	Rotating Scheimpflug (Sirius)	SIb	90.8	NA	86.2	84.9
Toprak [51]	2021	AS-OCT (MS-39)	Epith/Stromal thickness ratio	NA	NA	94	98.5
Peyman [73]	2023	NCT/Scheimpflug (Corvis ST)	TBI	85.8	> 0.33	78.08	76.81
Randleman [78]	2023	MT Brillouin microscopy	Mean plateau	100	< 5.696	100	100
			Minimum plateau	100	< 5.679	100	100
			Mean anterior 150 μm	100	< 5.702	100	100
			Minimum anterior 150 μm	100	< 5.684	100	100
Heidari [52]	2024	AS-OCT (Spectralis)	CCT	78.2	548	75	64.1
**Suspect KC vs.** **Normal**							
Scuderi [38]	2021	AS-OCT (RTVue)	C1–C2	98.5	NA	94.74	94
Salman [23]	2022	Rotating Scheimpflug (Sirius)	SIb	86	0.12	NA	84.66
Salman [87]	2022	Rotating Scheimpflug (Sirius)	RMS coma (3, ± 1)	92.2	> 0.2	95.24	75

*AUROC* = the area under the receiver operating characteristic curves; *FFKC* = forme fruste keratoconus; *SCKC* = subclinical keratoconus; *KC* = keratoconus; *AS-OCT* = anterior segment optical coherence tomography; *HOA* = higher-order aberrations; *As/Ps* = anterior/posterior corneal surface areas ratio; *BAD-D* = Belin-Ambrósio enhanced ectasia display deviation; *PE* = posterior elevation; *BFTE* = best-fit toric ellipsoid; *NCT* = non-contact tonometry; *TBI* = tomographic/biomechanical index; *KVf* = keratoconus vertex front; *PSD* = pattern standard deviation; *EEI-MAX* = maximum epithelium ectasia index; *BEI-MAX* = maximum Bowman’s layer ectasia index; *MPE* = maximum posterior elevation; *BFTA* = best-fit toric aspheric surface; *MCT* = minimal corneal thickness; *BCVf* = Baiocchi Calossi Versaci front; *MT* = motion tracking; *SIb* = symmetry index back; *CCT* = central corneal thickness; *RMS* = root mean square

## Conclusions

Despite the array of corneal imaging techniques available for diagnosing established KC, detecting early cases of this disease presents a significant challenge. The accuracy of screening and diagnosis can be improved through the synergistic application of multiple techniques. In instances where KC is suspected, even in the absence of apparent abnormalities in one particular technique, clinicians are advised to employ additional diagnostic modalities, either independently or in conjunction. This approach proves beneficial for identifying patients at an earlier stage and reduces the risk of potential postrefractive ectasia. Ongoing advancements in these diagnostic modalities promise to contribute to a more comprehensive understanding and improved management of KC.

## Data Availability

Not applicable.

## Abbreviations

AAI: Asphericity asymmetry index
ARTh: Ambrósio relational thickness to the horizontal profile
ARTmax: Ambrósio relational thickness maximum
AS-OCT: Anterior segment optical coherence tomography
AST: Keratometric astigmatism
BAD: Belin-Ambrosio enhanced ectasia display
BCV: Baiocchi Calossi Versaci
BFS: Best-fit sphere
BFTE: Best-fit toric ellipsoid
CBI: Corvis biomechanical index
CH: Corneal hysteresis
CRF: Corneal resistance factor
CSI: Center/surround index
DA: Amplitude of deformation
DAR: Deformation amplitude ratio
DSI: Differential sector index
FD-OCT: Fourier-domain optical coherence tomography
FFKC: Forme fruste keratoconus
HCR: Highest concavity radius
HOA: Higher-order aberration
IHA: Index of height asymmetry
IR: Integrated radius
I-S: Inferior-superior
ISV: Index of surface variance
IVCM: In vivo confocal microscopy
KC: Keratoconus
KISA: Keratoconus percentage index
KPI: Keratoconus prediction index
KSI: Keratoconus severity index
KVf: Keratoconus vertex front
KVb: Keratoconus vertex back
MT: Motion tracking
OCE: Optical coherence elastography
ORA: Ocular response analyzer
OSI: Opposite sector index
PRFI: Pentacam random forest index
PS-OCT: Polarization-sensitive optical coherence tomography
RMS: Root mean square
SCKC: Subclinical keratoconus
SD-OCT: Spectral-domain optical coherence tomography
SN-IT: Superonasal and inferotemporal
SPA1: Stiffness parameter at the first applanation
SRAX: Steepest radial axes
SIb: Symmetry index back
SIf: Symmetry index front
TBI: Tomographic/biomechanical index
TCT: Thinnest corneal thickness
TD-OCT: Time-domain optical coherence tomography
UHR-OCT: Ultrahigh-resolution optical coherence tomography
